# Supplementary material for: Cavity control of multiferroic order in single-layer NiI2
Source: NPJ Comput Mater. 2026 Jun 11;12(1):205. doi: 10.1038/s41524-026-02184-w (PMC13259918; doi:10.1038/s41524-026-02184-w)
Supplement: Supplementary file 1 — Supplementary Information [file 41524_2026_2184_MOESM1_ESM.pdf]

Supplementary Material for:
Cavity control of multiferroic order in single-layer NiI_2

Chongxiao Fan,[1,2,*] Emil Viñas Boström,[1,3,*] Xinle Cheng,[1] Lukas Grunwald,[1,2] Zhuquan Zhang,[4] Dante M. Kennes,[2,1] Dmitri N. Basov,[4] and Angel Rubio[1,5,3,†]

[1] *Max Planck Institute for the Structure and Dynamics of Matter, Luruper Chaussee 149, 22761 Hamburg, Germany*
[2] *Institute for Theory of Statistical Physics, RWTH Aachen University, and JARA Fundamentals of Future Information Technology, 52062 Aachen, Germany*
[3] *Nano-Bio Spectroscopy Group and ETSF, Departamento de Polímeros y Materiales Avanzados: Fisica, Química y Tecnologia, Universidad del País Vasco UPV/EHU- 20018 San Sebastián, Spain*
[4] *Department of Physics, Columbia University, New York, NY 10027, USA*
[5] *Initiative for Computational Catalysis, Flatiron Institute, Simons Foundation, New York City, NY 10010, USA*

(Dated: May 4, 2026)

Analytic derivation of the spin model

To supplement the numerical results, we also derive an analytical expression for the spin parameters J_1 and J_3 using a simplified model. We assume the hopping processes mediated by the ligands can be subsumed into an effective Ni-Ni hopping, thereby defining the effective hopping matrix

$$T_{ij} = \begin{pmatrix} t_1 & t_3/\sqrt{2} \\ t_3/\sqrt{2} & t_2 \end{pmatrix}. \tag{S1}$$

To second order in the hopping, the effective spin Hamiltonian is then given by

$$H_s = \sum_{\langle ij\rangle} \mathcal{P}\Phi^\dagger_{i\sigma} T_{ij}\Phi_{j\sigma}\mathcal{Q}\left[\frac{1}{E_0 - H_0}\right]\mathcal{Q}\Phi^\dagger_{j\sigma'}T_{ji}\Phi_{i\sigma'}\mathcal{P}. \tag{S2}$$

Here $\mathcal{P}$ is the projection operator onto the low-energy $S = 1$ manifold, $\mathcal{Q} = 1 - \mathcal{P}$ is the projector onto the complement, $E_0 = 2E_t = 2U - 6J_H$ is the energy of the Ni triplet state, and $H_0 = H_U$.

To derive the spin Hamiltonian, we evaluate the matrix elements of Eq. S2 in the local spin eigenstates $|m_1 m_2\rangle = |S_1 m_1, S_2 m_2\rangle$. The labels S_1 and S_2 can be suppressed since we always have $S_1 = S_2 = 1$. Starting from a configuration with two holes on each Ni, the intermediate state will always be a product of a one-hole state and a three-hole state, and have the energy $E = E_1 + E_3 = 3U - 5J_H$. We therefore have $E_0 - E = -U - J_H$, and within the triplet subspace we can write

$$H_s = \frac{-1}{U + J_H}\sum_{\langle ij\rangle}\mathcal{P}\Phi^\dagger_{i\sigma}T_{ij}\Phi_{j\sigma}\mathcal{Q}\mathcal{Q}\Phi^\dagger_{j\sigma'}T_{ji}\Phi_{i\sigma'}. \tag{S3}$$

To work out the matrix elements, we note that the Hamiltonian has the structure $H_{s,ij} = M^\dagger_{ji}M_{ji} + M^\dagger_{ij}M_{ij}$, where M_{ji} is a 9×16 matrix that takes a given triplet state $|m_1 m_2\rangle$ into state expressible in the product basis $|a_m b_n\rangle$, where $|a_m\rangle$ is a one-hole state and $|b_n\rangle$ is a three-hole state. Evaluating these matrices we find an effective spin Hamiltonian

$$H_s = \sum_{\langle ij\rangle}\mathbf{S}_i J_{ij}\mathbf{S}_j, \tag{S4}$$

where J_{ij} is the Heisenberg-like magnetic exchange with isotropic and anisotropic terms. For the simplified model considered here, we find with $t^2_{\text{eff}} = -2(t_1^2 + t_2^2 + t_3^2)$ that the effective exchange coupling is

$$J_i = \frac{t^2_{\text{eff}}}{U + J_H}. \tag{S5}$$

Cavity light-matter coupling

We now consider the effect of electromagnetic vacuum fluctuations on the equilibrium magnetic state of NiI_2. In presence of an electromagnetic field, the total Hamiltonian is given by $H = H_U + H_t + \sum_\lambda \hbar\Omega_\lambda \hat{n}_\lambda$, where $\hbar\Omega_\lambda$ is the energy of cavity mode λ, and $\hat{n}_\lambda$ is the corresponding number operator. Because of fluctuations of the electromagnetic field, the hopping amplitudes acquire an additional phase, and the Hamiltonian H_t is modified by the replacement

$$\hat{c}^\dagger_{i\alpha\sigma}\hat{c}_{i\beta\sigma} \to e^{i\phi_{ij}}\hat{c}^\dagger_{i\alpha\sigma}\hat{c}_{i\beta\sigma}. \tag{S6}$$

Here $\phi_{ij} = (ea/\hbar)\,\mathbf{r}_{ij}\cdot\hat{\mathbf{A}}$ is a Peierls phase, $\mathbf{r}_{ij} = \mathbf{r}_j - \mathbf{r}_i$ is the vector between atomic sites i and j (measured in units of the Ni-Ni distance a), and the quantum vector potential is

$$\hat{\mathbf{A}} = \sum_\lambda (A_\lambda \mathbf{e}_\lambda \hat{a}^\dagger_\lambda + A^*_\lambda \mathbf{e}^*_\lambda \hat{a}_\lambda). \tag{S7}$$

* These authors contributed equally to this work. emil.bostrom@mpsd.mpg.de
† angel.rubio@mpsd.mpg.de

Here A_λ is a mode function, and for a given mode λ this scheme defines the dimensionless light-matter coupling $g_\lambda = (ea/\hbar)A_\lambda = ea/\sqrt{2\epsilon_0\hbar\Omega_\lambda V}$. The Peierls phases can be written as in terms of dimensionless variables as $\phi_{ij} = \mathbf{r}_{ij}\cdot\hat{\mathbf{a}}$ with

$$\hat{\mathbf{a}} = \sum_\lambda (g_\lambda \mathbf{e}_\lambda \hat{a}_\lambda^\dagger + g_\lambda^* \mathbf{e}_\lambda^* \hat{a}_\lambda), \tag{S8}$$

and we note that ϕ_{ij} only depends on the positions of the ions involved. The modified hopping Hamiltonian and polarization operator is obtained by adding the appropriate Peierls phases to each of the bonds.

The total Hamiltonian can be expanded in the photon number basis $|\mathbf{n}\rangle = |n_1, n_2, \ldots, n_N\rangle$ according to [1]

$$\begin{aligned} H &= \sum_{\mathbf{nm}} (\mathbf{1}_e \otimes |\mathbf{n}\rangle\langle\mathbf{n}|) H (\mathbf{1}_e \otimes |\mathbf{m}\rangle\langle\mathbf{m}|) \\ &= \sum_{\mathbf{nm}} H_{\mathbf{nm}} \otimes |\mathbf{n}\rangle\langle\mathbf{m}|, \end{aligned} \tag{S9}$$

where $\mathbf{1}_e$ is the identity operator in the electronic Hilbert space, and the Hamiltonian $H_{\mathbf{nm}}$ is given by

$$H_{\mathbf{nm}} = \big(H_U + \sum_\lambda \hbar\Omega_\lambda n_\lambda\big)\delta_{\mathbf{nm}} + H_{t,\mathbf{nm}}. \tag{S10}$$

To calculate the matrix elements $\langle\mathbf{n}|e^{i\mathbf{d}_{ij}\cdot\hat{\mathbf{a}}}|\mathbf{m}\rangle$ we note that since $[\hat{a}_\lambda^\dagger, \hat{a}_{\lambda'}] = 0$, the Peierls phases factorize over different modes and

$$\langle\mathbf{n}|e^{i\mathbf{d}_{ij}\cdot\hat{\mathbf{a}}}|\mathbf{m}\rangle = \prod_\lambda \langle n_\lambda|e^{i\mathbf{d}_{ij}\cdot\hat{\mathbf{a}}_\lambda}|m_\lambda\rangle. \tag{S11}$$

The single-mode expectation values are calculated by introducing the variables $\eta_{ij\lambda} = g_\lambda(\mathbf{d}_{ij}\cdot\mathbf{e}_\lambda)$, and using the Baker-Hausdorff expansion for the exponential. The matrix elements are $\langle n_\lambda|e^{i\mathbf{d}_{ij}\cdot\hat{\mathbf{A}}_\lambda}|m_\lambda\rangle = i^{|n_\lambda - m_\lambda|} j^{ij}_{n_\lambda,m_\lambda}$, where explicit forms of $j^{ij}_{n_\lambda,m_\lambda}$ are provided in Ref. [1]. Using the notation $\mathbf{g} = \{g_1, g_2, \ldots, g_N\}$ to denote the set of couplings, the light-matter interaction is described by the function

$$J^{ij}_{\mathbf{nm}}(\mathbf{g}) = \langle\mathbf{n}|e^{i\mathbf{d}_{ij}\cdot\hat{\mathbf{A}}}|\mathbf{m}\rangle = \prod_\lambda j^{ij}_{n_\lambda,m_\lambda}. \tag{S12}$$

With this function, the hopping Hamiltonian in presence of the cavity can be written as

$$H_{t2,\mathbf{nm}} = -\sum_{\langle ij\rangle\sigma} \Phi^\dagger_{i\sigma} J^{ij}_{\mathbf{nm}}(\mathbf{g}) \begin{pmatrix} r_1 & r_3 \\ r_3 & r_2 \end{pmatrix} \Phi_{j\sigma}, \tag{S13}$$

here exemplified for a Ni-Ni bond.

Super-exchange origin of J_1 beyond the analytical model

To better understand the cavity-induced renormalization of the magnetic interactions, we analyze the behavior of the nearest-neighbor exchange J_1 in comparison with more conventional superexchange processes. For a single-orbital Hubbard model, the cavity modification can be obtained analytically and is given by

$$J_i(g,\omega) = J_{i,0}e^{-|g|^2} \sum_n \frac{|g|^{2n}}{n!}\frac{1}{1+\omega_s n}. \tag{S14}$$

To lowest order in $|g|^2$, this expression predicts a quadratic correction in the light-matter coupling, consistent with the generic form $X(g) = X_0 + |g|^2 X_1$. For standard super-exchange processes, or for straight (180°) bonds, the leading effect is a reduction of the exchange interaction with increasing g. This behavior is found, for example, for J_3, whose dominant contribution arises from direct Ni–Ni hopping.

The situation is different for the nearest-neighbor exchange J_1. According to the Goodenough–Kanamori rules, J_1 is mainly associated with the 90° Ni–I–Ni bond geometry and therefore originates primarily from ligand-mediated hopping processes. In this case, the simple analytic expression above is not directly applicable, since the dominant contributions involve higher-order virtual processes and a more complicated structure of both the effective hoppings and the energy denominators of intermediate states.

To clarify this point, we performed additional numerical calculations for a four-atom cluster consisting of two Ni and two I ions. Fig. S1 shows that the dominant contributions to both J_1 and K arise from ligand-mediated processes and increase with increasing light-matter coupling. By contrast, when only direct Ni–Ni hopping processes are retained, the exchange decreases with g, as expected from the simplified super-exchange picture. This demonstrates that the enhancement of J_1 and K originates from ligand-mediated contributions.

Surface phonon polaritons

The discussion above holds for any cavity mode structure, but we now specialize to a surface cavity where the electric field comes from surface phonon polaritons (SPP) of a paraelectric surface [2]. The electric field corresponding to the SPPs can be written as

$$\begin{aligned} \mathbf{E}_\parallel(\mathbf{x},t) &= -i\sum_{\mathbf{q}} f_q \frac{\mathbf{q}}{|q|} e^{-qd} e^{i\mathbf{q}\cdot\mathbf{x} - i\omega_s t} \left(\hat{a}^\dagger_{\mathbf{q}} + \hat{a}_{-\mathbf{q}}\right) \\ E_\perp(\mathbf{x},t) &= \sum_{\mathbf{q}} f_q e^{-qd} e^{i\mathbf{q}\cdot\mathbf{x} - i\omega_s t} \left(a^\dagger_{\mathbf{q}} + a_{-\mathbf{q}}\right) \\ f_q &= \sqrt{\frac{q\hbar(\omega_s^2 - \omega_{\mathrm{TO}}^2)}{4A\epsilon_0(\epsilon_{\mathrm{sub}} + \epsilon_{\mathrm{mat}})\omega_s}}, \end{aligned} \tag{S15}$$

where $\omega_s^2 = (1/2)(\omega_{\mathrm{LO}}^2 + \omega_{\mathrm{TO}}^2)$, and ω_{LO} and ω_{TO} are the longitudinal and transverse optical phonon frequencies of the substrate. For these modes, we can calculate the

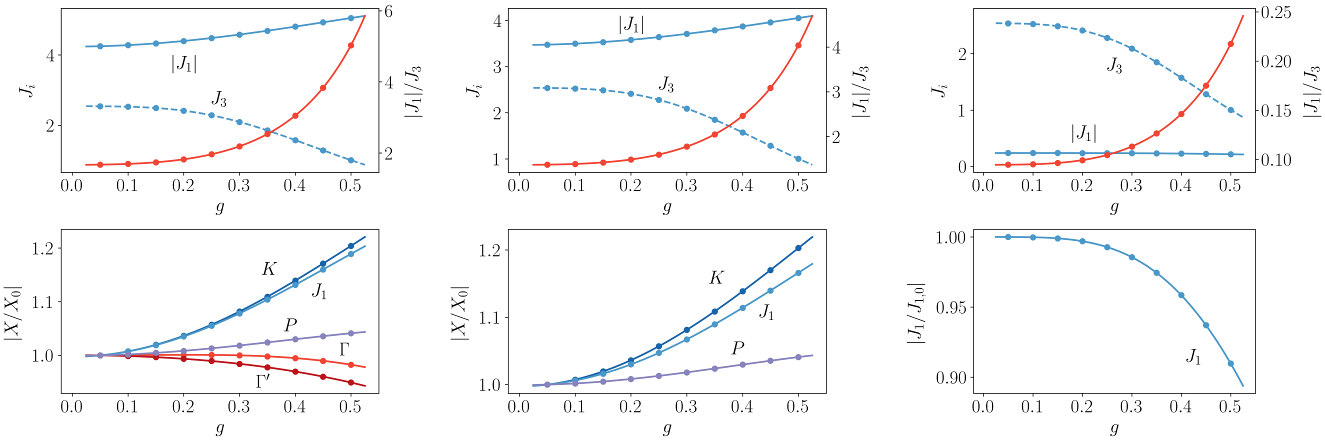

FIG. S1. Cavity renormalization of nearest neighbor magnetic interactions including (left) the full hopping structure, (center) only hopping over the I ligands, and (right) only direct Ni-Ni hoppings.

local electric field noise $\langle \mathbf{E}^2 \rangle$, which has the form [2]

$$\epsilon_0 \langle \mathbf{E}^2 \rangle = \frac{\hbar^2(\omega_s^2 - \omega_{\mathrm{TO}}^2)}{16\pi\hbar\omega_s \epsilon_r d^3}. \tag{S16}$$

As discussed in the main text, this can be used to construct a single effective mode approximation.

The SPP electric field can be compared with that at the center of a Fabry-Perot cavity, where the local electric field noise is $\epsilon_0\langle \mathbf{E}^2\rangle = 7\pi^2\omega_c/1080h^3$ [2]. Here $\omega_c = c\pi/h$ is the fundamental cavity frequency and h is the height of the cavity, which for a Fabry-Perot cavity are not independent. For a THz cavity we find $h \sim 10$ μm, while d is independent of frequency and can be taken as $d \sim 10$ nm, this leads to a large enhancement $\eta = \epsilon_0\langle \mathbf{E}^2\rangle_{\mathrm{SPP}}/\epsilon_0\langle \mathbf{E}^2\rangle_{\mathrm{FP}} \sim h^3/d^3 \sim 10^9$ of the effective electric field.

Analytic solution for the $J_1 - J_3 - A_{zz}$ model

To analytically estimate the ground state energy of the helical state in the macroscopic limit, we consider an effective $J_1 - J_3 - A_{zz}$ model on a triangular lattice. This is expected to provide a good approximation to the true ground state, since the full spin Hamiltonian is dominated by the competition between the J_1 and J_3 terms. We compare the energies of two helical magnetic states, whose propagation vectors are along the $[1\bar{1}0]$ and $[100]$ directions, and the ferromagnetic state.

For each classical spin $\mathbf{S}_i$, with length $|\mathbf{S}_i| = 1$, the next spin along the propagation direction is obtained by the rotation $\mathbf{S}_{i+1} = R(\theta, \varphi, \alpha)\mathbf{S}_i$, while the previous one is $\mathbf{S}_{i-1} = R(\theta, \varphi, -\alpha)\mathbf{S}_i$. The rotation matrix $R(\theta, \varphi, \alpha)$ is defined by the polar and azimuthal angles θ and φ of the normal vector of the spin rotational plane, and the relative angle $\alpha \in [0, \pi]$ within this plane. If $\alpha = 0$, the system is in the ferromagnetic phase. With a positive single-ion anisotropy A_{zz}, a finite out-of-plane component S_z will increase the energy of the magnetic state, and so the ground state is described by an effective XY model with $\theta = 0$. The Hamiltonian of the single helix states with $\mathbf{q}_1 \parallel [1\bar{1}0]$ and $\mathbf{q}_2 \parallel [100]$ are then

$$H_{q_1} = N\Big[J_1(1 + 2\cos\alpha) + J_3\,(1 + 2\cos 2\alpha)\Big], \tag{S17}$$

$$H_{q_2} = N\Big[J_1(2\cos\frac{\alpha}{2} + \cos\alpha) + J_3(2\cos\alpha + \cos 2\alpha)\Big]. \tag{S18}$$

In NiI_2 the nearest neighbor interaction is ferromagnetic, $J_1 < 0$, while the third nearest neighbor is antiferromagnetic, $J_3 > 0$, and we denote their ratio by $\eta = |J_1|/J_3 > 0$. To ease the notation we write $x_1 = \cos\alpha \in [-1, 1]$ and $x_2 = \cos(\alpha/2) \in [-1, 1]$, such that the Hamiltonian is

$$H_{q_1} = NJ_3(4x_1^2 - 2\eta x_1 - \eta - 1), \tag{S19}$$

$$H_{q_2} = NJ_3\left[8x_2^4 - (2\eta + 4)x_2^2 - 2\eta x_2 + \eta - 1\right]. \tag{S20}$$

For Eq. S19, the minimum occurs at

$$x_1 = \begin{cases} \frac{\eta}{4}, & \eta \in (0, 4] \\ 1, & \eta \in (4, +\infty) \end{cases} \tag{S21}$$

For Eq. S20, the minimum occurs at

$$x_2 = \begin{cases} \frac{1}{4}(1 + \sqrt{1 + 2\eta}), & \eta \in (0, 4] \\ 1, & \eta \in (4, +\infty) \end{cases} \tag{S22}$$

In both cases, the system transitions from a ferromagnetic to a helimagnetic state at $\eta = 4$, or $|J_1| = 4J_3$. We can also determine the more stable helical phase by comparing the ground state energies E_{0,q_i} of the Hamiltonian H_{q_1} and H_{q_2}. Their ground state energies are

$$E_{0,q_1} = NJ_3\left(-\eta - 1 - \frac{\eta^2}{4}\right), \tag{S23}$$

$$E_{0,q_2} = \frac{NJ_3}{8}(-4\eta\sqrt{1 + 2\eta} + 2\eta - 2\sqrt{1 + 2\eta} - 10 - \eta^2). \tag{S24}$$

We find that $E_{0,q_2} \leq E_{0,q_1}$ for all $\eta \in (0,4]$, showing that the helix order prefers to propagate along [100] direction.

Furthermore, we also evaluated the spin stiffness for the preferred helix state, which is defined as

$$\rho_s = \frac{1}{N}\frac{\partial^2 H_{q_2}}{\partial \alpha^2}\Big|_{\alpha=\alpha_0}, \quad \text{(S25)}$$
$$= -2J_1 \cos\frac{\alpha_0}{2} - (4J_1 + 8J_3)\cos\alpha_0 - 16J_3\cos 2\alpha_0.$$

In Fig. S2, we show the theoretically determined α_0 and the corresponding classical spin stiffness ρ_s/k_B as functions of the distance d to the surface cavity. Near the critical distance $d_c \approx 1.6$ nm, the stiffness approaches zero, indicating a softening of the stretching mode and an enhanced sensitivity to quantum fluctuations in this regime. A more comprehensive understanding of the physics in the vicinity of d_c would require going beyond a classical description and thermal fluctuations, and to analyze the corresponding quantum spin model on the triangular lattice.

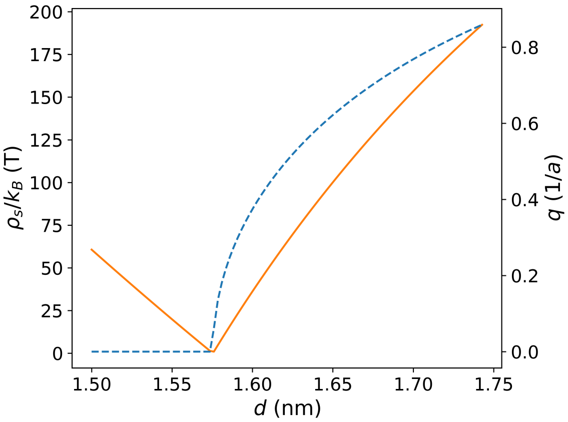

FIG. S2. Theoretically calculated spiral momentum q (blue dashed line), related to α_0, and the classical spin stiffness ρ_s/k_B (orange solid line) of NiI_2.

Specific heat

From the Monte Carlo simulations, we obtain the specific heat per spin $C_v(T)$ as a function of distance d and temperature T. It is defined as

$$C_v(T) = \frac{\langle E^2\rangle - \langle E\rangle^2}{N k_B T^2}, \quad \text{(S26)}$$

where E is the measured total energy. The results in Fig. S3 show a large specific heat at large d, for temperature $T \approx 12.8$ K, and a finite size scaling consistent with a second order phase transition from a paramagnetic to a helimagnetic phase. Compared with Fig. 3c of the main text, the phase boundaries are similar, apart from the low temperature transition from the HM and FM phases. From an energy perspective, these transitions show a smooth crossover rather than a second order phase transition. Together with the spin stiffness, these findings show that topological defects enrich the nature of these transitions, making the meron phase worthy of further study.

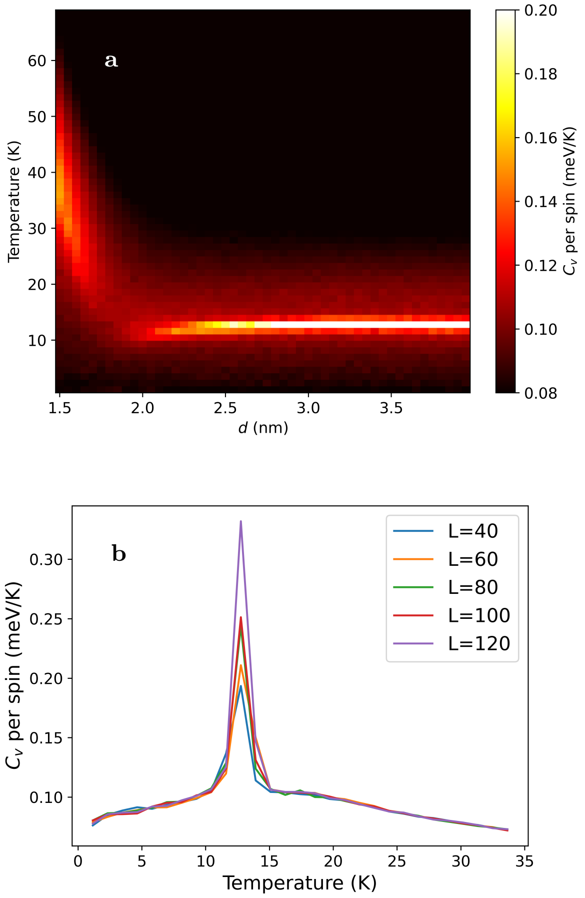

FIG. S3. **a,** Simulated specific heat per spin, C_v, as a function of distance d and temperature T. **b,** Simulated specific heat per spin, C_v, as a function of temperature T for different system sizes L. The distance is chosen as $d = 3.95$ nm.

[1] E. Viñas Boström, A. Sriram, M. Claassen, and A. Rubio, npj Computational Materials **9** (2023), 10.1038/s41524-023-01158-6.

[2] E. Viñas Boström, M. H. Michael, C. Eckhardt, and A. Rubio, Phys. Rev. Res. **7**, 033163 (2025).
